# m6A modification: recent advances, anticancer targeted drug discovery and beyond

**DOI:** 10.1186/s12943-022-01510-2

**Published:** 2022-02-14

**Authors:** Li-Juan Deng, Wei-Qing Deng, Shu-Ran Fan, Min-Feng Chen, Ming Qi, Wen-Yu Lyu, Qi Qi, Amit K. Tiwari, Jia-Xu Chen, Dong-Mei Zhang, Zhe-Sheng Chen

**Affiliations:** 1 Formula-pattern Research Center, School of Traditional Chinese Medicine, Jinan University, Guangzhou, China; 2 Guangdong Province Key Laboratory of Pharmacodynamic Constituents of Traditional Chinese Medicine and New Drugs Research, College of Pharmacy, Jinan University, Guangzhou, 510632 China; 3 Clinical Translational Center for Targeted Drug, Department of Pharmacology, School of Medicine, Jinan University, Guangzhou, 510632 People’s Republic of China; 4 Department of Pharmacology and Experimental Therapeutics, The University of Toledo, Toledo, OH USA; 5 Department of Pharmaceutical Sciences, College of Pharmacy and Health Sciences, St. John’s University, Queens, NY 11439 USA

**Keywords:** m6A, Cancer, Modulators, Drug discovery, Natural product, Chemosynthesis

## Abstract

Abnormal N6-methyladenosine (m6A) modification is closely associated with the occurrence, development, progression and prognosis of cancer, and aberrant m6A regulators have been identified as novel anticancer drug targets. Both traditional medicine-related approaches and modern drug discovery platforms have been used in an attempt to develop m6A-targeted drugs. Here, we provide an update of the latest findings on m6A modification and the critical roles of m6A modification in cancer progression, and we summarize rational sources for the discovery of m6A-targeted anticancer agents from traditional medicines and computer-based chemosynthetic compounds. This review highlights the potential agents targeting m6A modification for cancer treatment and proposes the advantage of artificial intelligence (AI) in the discovery of m6A-targeting anticancer drugs.

**Graphical abstract:**

Three stages of m6A-targeting anticancer drug discovery: traditional medicine-based natural products, modern chemical modification or synthesis, and artificial intelligence (AI)-assisted approaches for the future. 
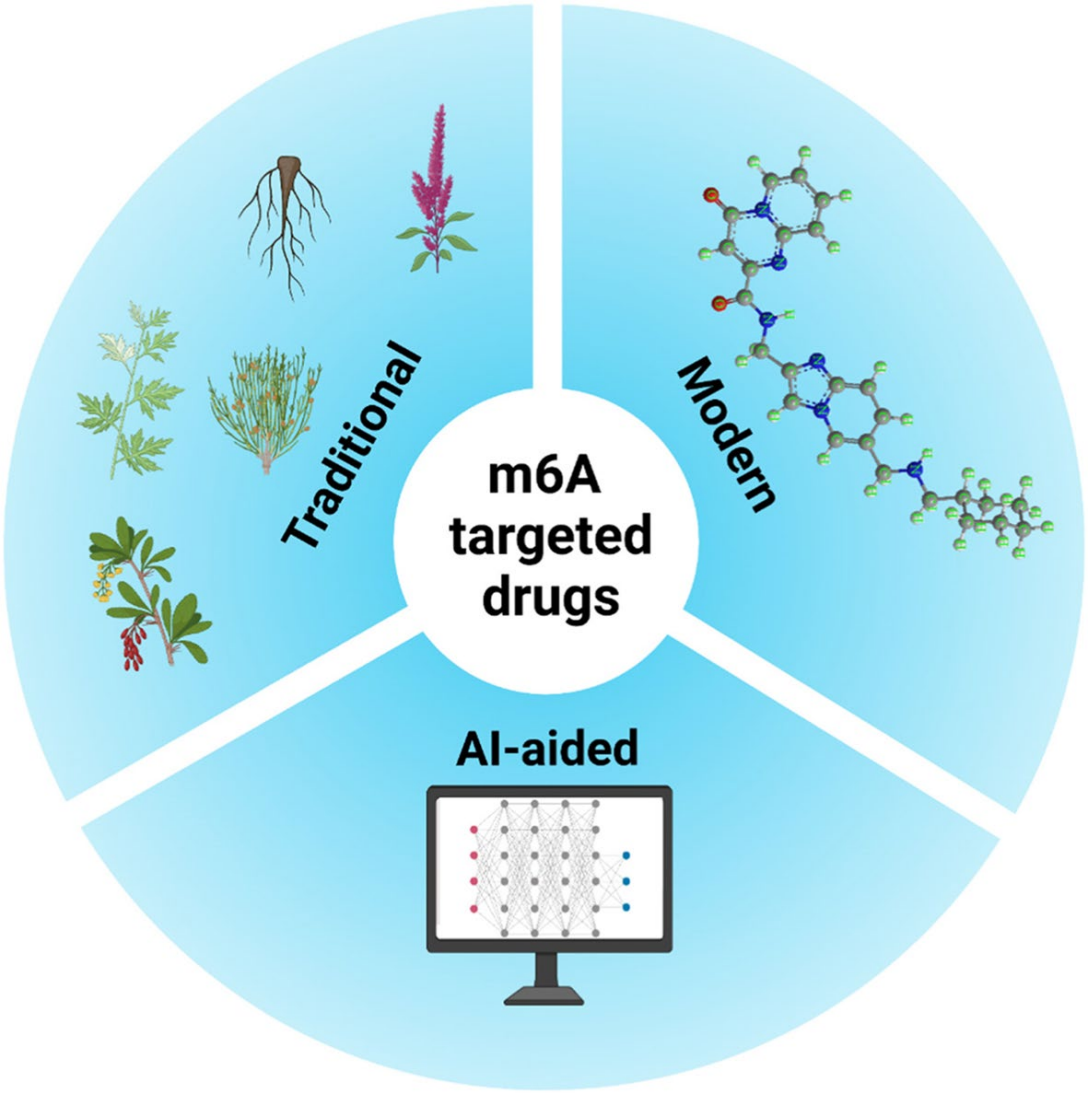

## Introduction

More than 170 types of posttranscriptional RNA modifications have been discovered since the 1960s [1]. Among them, N6-methyladenosine (m6A) is the most common modification in eukaryotic messenger RNAs (mRNAs) [1]. Accumulating evidence has demonstrated that m6A modification plays a critical role in regulating RNA processing, splicing, nucleation, translation, and stability, which is crucial for the development of multiple human diseases, such as cancer [2]. m6A modification is a dynamic and reversible process regulated by methylases (“writers”) and demethylases (“erasers”). The “writers” consist of a complex including methyltransferase-like 3 (METTL3), METTL14, METTL16, RNA-binding motif protein 15 (RBM15) and its paralogue RBM15B, zinc finger CCCH-type containing 13 (ZC3H13), vir-like m6A methyltransferase-associated protein (VIRMA, also named KIAA1429), and Wilms tumor 1 associated protein (WTAP), which are responsible for transferring methyl groups from the donor S-adenosylmethionine (SAM) to adenine [3–5]. Among them, METTL3, METTL14, and WTAP are the core members of this complex [5]. Then, m6A methylation is recognized by binding protein “readers”, such as YTH domain family proteins, insulin-like growth factor 2 mRNA-binding proteins (IGF2BPs), and heterogeneous nuclear ribonucleoprotein (HNRNP) family proteins [6]. The reversible process of m6A demethylation is also facilitated by “erasers”, such as fat mass and obesity-related protein (FTO) and alkB homologue 5 (ALKBH5) [3]. An increasing body of evidence shows that the “writer” METTL3, “eraser” FTO, and “reader” YTH domain families are involved in various stages of many types of hematomas and solid tumors and could be promising targets for anticancer therapy.

Continuous efforts are being made to discover highly effective and safe lead compounds targeting m6A modification [7–9]. Traditional medicine-based natural products, which have novel structures, multiple biological activities, and proven safety [10–15], are considered a valuable resource for drug discovery of m6A modulators. Modern drug discovery platforms, which are characterized by the integration of omics data, network pharmacology, natural resource-derived chemical databases, computer-aided design, and chemical modifications, have recently been applied to drug discovery [16–19]. Notably, this approach can effectively avoid the waste of experimental raw materials and laborious efforts, which will help to further evolve the discovery process of m6A-targeting drugs.

Although the physiological roles of m6A modulation in the development and progression of cancer have been wildly studied and some review articles related to m6A have been published [2, 3, 20], an update on the academic progress of m6A modulation is still necessary since many new related findings have been described recently. Here, we summarize the advances in m6A modulation and the core function of segments of m6A modulators in cancer. We also summarize the discovery of m6A-targeting anticancer agents from traditional medicine-based natural products and the use of a combination of artificial intelligence (AI) and chemosynthesis for drug exploration.

## Molecular composition of the m6A RNA methylation regulators

**Fig. 1:**
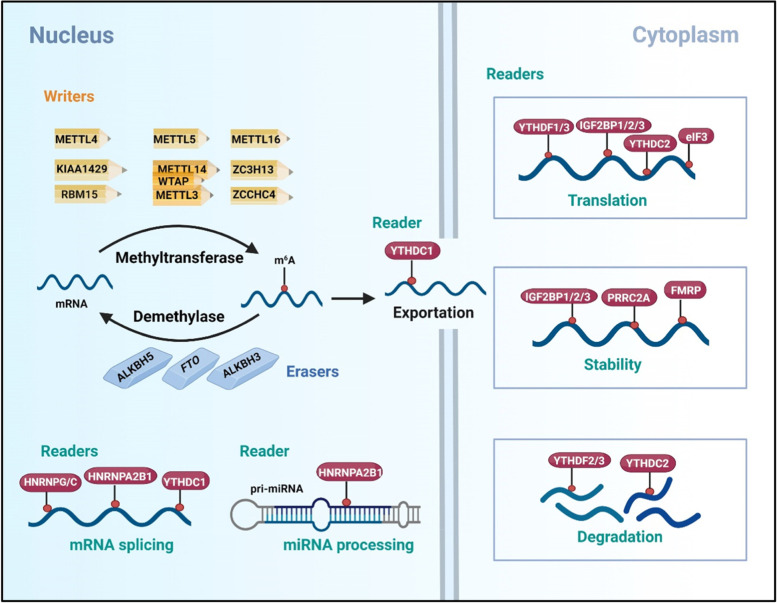
The underlying mechanisms of m6A modification. The m6A modification of mRNA is mainly catalyzed by the core methylase complex METTL3-WTAP-METTL14. RBM15/15B, VIRMA/KIAA1429, and ZC3H13 are newly identified mRNA m6A writers; METTL4, and METTL16 are snRNA m6A writhers; and METTL5 and ZCCHC4 are rRNA m6A writers. The m6A modification is removed by FTO, ALKBH5, and ALKBH3. Readers recognize m6A and affect various functions of RNAs, and they mainly include members of the YTH domain-containing family, the IGF2BP family, the HNRNP family, eIF3, PRRC2A, and FMRP

The m6A methylation of RNAs has been revealed to regulate numerous steps throughout the RNA life cycle, such as RNA splicing, decay/degradation, nuclear export, stability, and translation (Fig. 1) [20]. The molecular composition of the m6A RNA methylation regulators includes m6A methyltransferases, m6A demethylases, and m6A recognition factors. m6A methyltransferases, also called “m6A writers”, contain METTL3, METTL14 [21], and WTAP [22]. METTL3, METTL14 and WTAP form a complex and can anchored to the nucleus to catalyze m6A methyltransferase [3–5]. As METTL3 and METTL14 are the predominant m6A methyltransferases on mRNA, we mainly focus on METTL3/METTL14-mediated RNA m6A modification in this review. Additionally, it should be noted that other multicomponent methyltransferase complexes have recently been discovered and characterized, such as RBM15/RBM15B, VIRMA (KIAA1429), and ZC3H13. RBM15/15B, which interacts with WTAP and METTL3, has been identified as an additional component of the m6A methylation complex [2, 23]. VIRMA (KIAA1429) is associated with the methylation complex METTL3/METTL14/WTAP and cooperatively regulates m6A modification [24]. ZC3H13 anchors WTAP in the nucleus to enhance m6A modification [25]. Moreover, there are another identified m6A methyltransferases, including METTL16, METTL4, METTL5, and zinc finger CCHC-type containing 4 (ZCCHC4). The binding sites of METTL16 revealed no overlap with those of METTL3/METTL14 methylation complex [26], and METTL16 can mediate the m6A methylation of U6 snRNA, noncoding RNAs, and precursor mRNAs (pre-mRNAs) [27]. ZCCHC4 is an m6A methyltransferase of 28S rRNA [28] and METTL5 can induce the m6A methylation of 18S rRNA [29]. METTL4 mediates the m6A methylation of U2 snRNA to regulate pre-mRNA splicing [30].

Dynamic m6A methylation can be reversed by m6A demethylases in nucleus, also called “m6A erasers”, including FTO and ALKBH5. The demethylation of m6A modification in nucleic acids by FTO relies on the oxidative function of FTO in an Fe(II)- and α-KG-dependent manner [31]. ALKBH5 is another m6A demethylase that regulates the export and metabolism of mRNA by demethylating m6A modification [32]. In addition, members of the Alkb subfamily, such as ALKBH3, are responsible for removing m6A modification on tRNA [33].

The m6A recognition factor is known to regulate mRNA splicing, nuclear export, decay/degradation, translation, and stability and is also called an “m6A readers”. The YTH domain-containing family includes YTHDF1, YTHDF2, and YTHDF3, which are cytosolic m6A readers that regulate m6A degradation and translation [34]. YTHDF1 is reported to play a vital role in promoting translation in the cytosol, whereas YTHDF2 regulates mRNA degradation by mediating the lifetime of target transcripts [35, 36]. Nonetheless, gene expression, cell death, and survival are associated with the YTHDF2-mediated RNA process [37, 38]. YTHDF3 cooperates with YTHDF1 and YTHDF2 to affect the translation and decay of m6A-decorated mRNA, and inversely regulates their function [39]. Another YTH domain-containing family YTHDC1 regulates RNA nuclear export [40] and splicing [41], while YTHDC2 modulates the translation and abundance of target genes [42]. IGF2BPs include IGF2BP1, IGF2BP2, and IGF2BP3, and they primarily promote the stability and translation of target mRNAs [43]. The RNA-binding protein fragile X mental retardation protein (FMRP), encoded by the fragile X mental retardation 1 gene (FMR1), can promote the nuclear export [44] and stability [45] of m6A-modified RNAs. Eukaryotic initiation factor 3 (eIF3) preferentially binds to m6A-makred mRNA rather than nonmethylated RNA and is associated with the process of mRNA translation [46]. HNRNP family contains HNRNPA2B1, HNRNPC, and HNRNPG [6]. HNRNPA2B1 recognizes m6A-marked primary miRNAs (pri-miRNAs) and stimulates miRNA processing [47], while HNRNPC recognizes m6A to induce splicing in mRNA secondary structures [48]. Proline rich coiled-coil 2A (PRRC2A) is a novel m6A reader that can bind to a consensus GGACU motif in the *Olig2* coding sequence to stabilize *Olig2* mRNA [49].

## Aberrant m6A functions and tumor progression

**Fig. 2:**
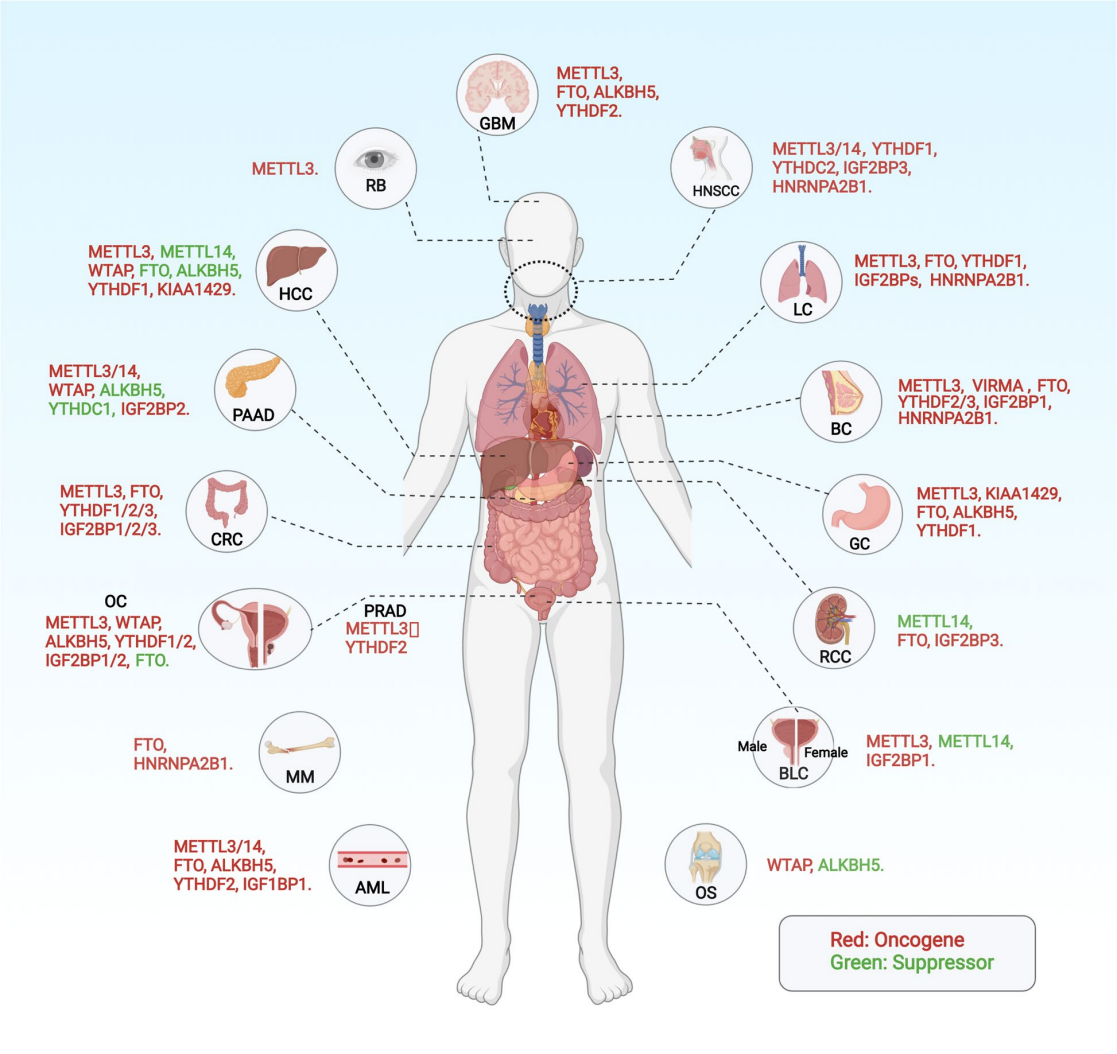
The role of m6A in human cancers. m6A modification regulators affect the progression of different types of human cancers by functioning as either tumor promoters or tumor suppressors. Abbreviations: AML, Acute myeloid leukemia; BLC, Bladder cancer; BC, Breast cancer; CRC, Colorectal cancer; GC, Gastric cancer; GBM, Glioblastoma; HCC, Hepatocellular carcinoma; HNSCC, Head and neck squamous cell carcinoma; MM, Multiple myeloma; LC, Lung carcinoma; OS, Osteosarcoma; OC, Ovarian cancer; PAAD, Pancreatic adenocarcinoma; PRAD, Prostate adenocarcinoma; RB, Retinoblastoma; RCC, Renal cell carcinoma

m6A modification is aberrant in various types of cancer and is associated with patient prognosis. The dysregulation of m6A modification also critically regulates malignant behaviors, such as proliferation, metastasis, tumor stemness, and drug resistance [50–53]. It has been reported that m6A modification regulators can function as either tumor promoters or tumor suppressors in different tumor types. The roles of m6A modification regulators in different types of cancer are summarized in Fig. 2.

m6A modification regulators affect the pathogenesis and progression of tumors via various mechanisms. When a m6A modulator acts as a tumor promotor, it promotes tumor progression by upregulating oncogenes or downregulating tumor suppressor genes. In contrast, when a m6A modulator functions as a tumor suppressor, it inhibits tumor progression by suppressing the expression of oncogenes or upregulating tumor suppressor genes. The effects and mechanisms of m6A modification regulators on tumor progression are summarized in Table 1.

**Table 1:** The effects and mechanisms of m6A modification regulators on tumor progression

Cancer Type	Type	m6A regulator	Related factor	Function	Ref.
AML	Writer	METTL3	CEBPZ, SP1	Maintains the leukemic state	[54]
			*C-MYC, BAL2, PTEN*	Inhibits differentiation and increases cell growth	[55]
		METTL14	*MYB, MYC*	Inhibits myeloid differentiation and enhance self-renewal of leukemia stem/initiation cells	[56]
	Eraser	FTO	*ASB2, RARA*	Promotes cell transformation and leukemogenesis, and inhibits leukemia cell differentiation	[57]
			*MYC, CEBPA*	Induces tumorigenesis	[58]
			*LILRB4*	Maintains cancer stem cell self-renewal and contributes to immune evasion	[59]
		ALKBH5	*TACC3*	Contributes to poor prognosis, maintenance of AML and self-renewal of leukemia stem/initiating cells	[60]
	Reader	YTHDF2	*Tnfrsf2*	Maintenance of leukemic stem cells	[61]
		IGF2BP1	LIN28B, let-7a	Enhances tumorigenicity	[62]
BC	Writer	METTL3	HBXIP, let-7 g	Accelerates cell proliferation in BC and promotes cancer progression	[63]
			P21	Contributes to worse prognosis and shorter disease-free survival and promote proliferation of cancer cell	[64]
			Adenylate kinase 4 (AK4)	Contributes to tamoxifen resistance	[65]
			Pri-mi-221-3p	Promotes adriamycin resistance	[66]
			ERRγ, *ESRRG*	Induces chemoresistance of cancer cell	[67]
	Eraser	FTO	*BNIP3*	Contributes to poor prognosis, promotes cancer cell proliferation, colony formation and metastasis	[68]
			MiR-181b-3p, ARL5B	Promotes invasion and migration of cancer cell	[69]
	Reader	YTHDF2	MYC	Upregulated in TNBC, depletion of YTHDF2 suppresses tumor growth, triggers activation of EMT, initiate apoptosis, and sensitizes TNBC cells to proteotoxic	[37]
		YTHDF3	*ST6GALNAC5, GJA1, EGFR*	Contributes to breast cancer brain metastasis and poor survival	[70]
		KIAA1429	CDK1	Contributes to shorter overall survival of patients and promotes cancer cell proliferation and metastasis	[71]
		IGF2BP1	LncRNA KB-1980E6.3, *c-Myc*	Maintains the stemness of breast cancer cells and tumorigenesis	[72]
		HNRNPA2B1	Erα, miR-222-3p	Induces acquired endocrine-resistance	[73]
BLC	Writer	METTL3	Pri-miR221/222, PTEN	Contributes to poor prognosis of BLC patients and promotes tumor cell proliferation	[74]
			*AFF4, IKBKB, RELA, MYC*	Promotes cancer progression	[75]
		METTL14	*Notch1*	Inhibits bladder tumor initiating cells self-renewal and bladder tumorigenesis	[76]
	Reader	IGF2BP1	MYC, FSCN1, circPTPRA	Promotes BLC growth and aggressiveness in vivo and in vitro	[77]
CRC	Writer	METTL3	MiR-1246, SPRED2, MAPK pathway	Contributes to tumor metastasis	[78]
			GLUT1, mTORC1 signaling	Contributes to poor survival and promote CRC initiation and progression	[79]
			*SOX2*	Contributes to poor prognosis, cell self-renewal, stemness, migration, tumorigenesis and metastasis	[80]
			*HK2, SLC2A1*	Drives glycolytic metabolism, promotes tumorigenesis	[81]
			Circ1662	Promotes CRC cell invasion and migration	[82]
		METTL14	Pri-miR-375	Inhibits CRC cell growth and metastasis	[83]
			*SOX4*	Inhibits CRC cells migration, invasion and metastasis	[84]
			LncRNA XIST	Suppresses proliferation and metastasis	[51]
		METTL3/METTL14	*STAT1, IRF1*	Regulates immune responses to anti-PD-1 therapy	[85]
	Eraser	FTO	MYC	Promotes cancer progression	[86]
	Reader	YTHDF1	FZD9, WNT6	Promotes tumorigenicity and regulates stem cell-like activity	[87]
		YTHDF2	*GASK3β,* miRNA-6125	Promotes cancer growth	[88]
		YTHDF3	YAP, lncRNA GAS5	Contributes to poor overall survival, promotes CRC cell proliferation, invasion, metastasis	[89]
		IGF2BP1	RBRP, *c-Myc*	Promote tumorigenesis	[90]
		IGF2BP2	LINC00460, DHX9, *HMGA1*	Promote tumor progression	[91]
		IGF2BP3	*CCND1, VEGF*	Associates with cancer progression and survival, regulates cell cycle and angiogenesis	[92]
EC	Writer	METTL3/METTL14	PHLPP2, AKT, mTOR	Promotes cell proliferation and tumorigenicity	[93]
GBM	Writer	METTL3	*SRSF, BCL-X, NCOR2*	Promotes the growth and self-renewal of glioma stem cells	[94]
			*ADAR1, CDK2*	Contributes to tumorigenesis	[95]
			*SOX2,* HuR	Induces resistance to γ-irradiation and promotes DNA repair	[96]
	Eraser	FTO	–	Promotes glioma stem cell (GSC) growth and self-renewal	[97]
		ALKBH5	FOXM1	Enhances self-renewal and tumorigenesis of GBM stem-like cell	[98]
	Reader	YTHDF2	EGFR, SRC, ERK, LXRA, HIVEP2	Contributes to poor prognosis, promotes GBM cell proliferation, invasion, and tumorigenesis.	[99]
GC	Writer	METTL3	LncRNA ARHGAP5-AS1, *ARHGAP5*	Promotes chemoresistance	[100]
			*PTEN, TMEM127,* pri-miR-17–92	Contributes to poor prognosis and enhance sensitivity to everolimus	[101]
			*HDGF*	Contributes to poor prognosis, promote cancer cell proliferation, liver metastasis, angiogenesis, glycolysis	[102]
			*BATF2*	Promotes tumor progression and metastasis	[103]
			*ZMYM1*	Promotes EMT program and metastasis	[104]
		KIAA1429	*C-Jun*	Promotes cancer cell proliferation	[105]
	Eraser	FTO	–	Promotes proliferation and migration of cancer cell	[106]
		ALKBH5	LncRNA NEAT1	Promotes invasion and metastasis	[107]
	Reader	YTHDF1	*FZD7*	Contributes to aggressive tumor progression and poor overall survival, promotes proliferation and tumorigenesis	[108]
HCC	Writer	METTL3	CTNNB1, Wnt/β-catenin pathway	Promotes tumor progression	[109]
			LINC00958	Promotes tumor progression	[110]
			*SOCS2*	Contributes to poor prognosis of patients with HCC, promotes HCC growth	[111]
		METTL14	DGCR8, miRNA 126	Inhibits tumor metastasis	[112]
		WTAP	HuR, p21/27, Ets-1	Contributes to poor prognosis and contributes to the progression of HCC	[113]
		KIAA1429	*GATA3*, lncRNA GATA3-AS	Contributes to poor prognosis, promote tumor growth and metastasis	[114]
	Eraser	FTO	SIRT1	Inhibits cancer tumorigenesis	[115]
		ALKBH5	LYPD1, IGF2BP1	Suppresses cancer cell proliferation and invasion	[116]
	Reader	YTHDF1	HIF-1α, *ATG2A, ATG14*	Contributes to poor prognosis, promotes autophagy and autophagy-related malignancy	[117]
			*EGFR*	Promotes cell viability and metastasis	[118]
		YTHDF2	MiR-145	Contributes to malignancy of HCC	[119]
			IL11, SERPINE2	Suppresses tumor growth, vasculature remodeling and metastasis	[120]
HNSCC	Writer	METTL3/METTL14	LNCAROD	Promote malignant development in HNSCC	[121]
		METTL3	CircCUX1	Contributes to radiotherapy resistance in HSCC	[122]
			*ZNF750*	Modulates NPC progression	[123]
			YTHDF1, *c-Myc*	Promotes the proliferation, invasion, migration tumor growth in OSCC progression	[124]
			BMI1	Contributes to poor prognosis, promotes OSCC proliferation, self-renewal, tumor growth and metastasis	[125]
		RBM15	*TMBIM6*, IGF2BP3	Contributes to unfavorable prognosis, promotes the proliferation, invasion, migration, and apoptosis of LSCC	[126]
	Reader	HNRNPA2B1	LINE-1, TGF-β1, Smad2, Slug	Contributes to poor overall survival, promotes OSCC tumorigenesis and metastasis	[127]
		YTHDC2	IGF1R, AKT, S6	Promote radiotherapy resistance in NPC	[128]
		YTHDF1	*TFRC*	Promote HSCC tumorigenesis	[129]
LC	Writer	METTL3	LncRNA LCAT3	Modulates LC progression	[130]
			MiR-143-3p	Promotes brain metastasis of LC	[131]
			MALAT1-miR-1914-3p-YAP axis	Contributes to drug resistance and metastasis	[132]
			TAZ, EGFR	Promotes LC growth, survival, and invasion	[133]
	Reader	IGF2BPs	CircNDUFB2	Promotes tumor progression and metastasis, modulates immune responses	[134]
		HNRNPA2B1	LncRNA 01234	Promote cancer cell growth and inhibit apoptosis	[135]
		YTHDF1	Keap1-Nrf2-AKR1C1 axis	Contributes to hypoxia adaptation and pathogenesis of NSCLC	[136]
MEL	Eraser	FTO	IFNγ, *PD-1, CXCR4, SOX10*	Promotes tumorigenesis and anti-PD-1 resistance	[137]
	Reader	YTHDF1	*HINT2*	Inhibits tumor progression	[138]
		YTHDF2	*PER1, TP53*	Accelerates tumorigenesis of ocular MEL	[139]
MM	Eraser	FTO	HSF1	Promotes MM proliferation, migration, and invasion	[140]
	Reader	HNRNPA2B1	AKT3, ILF3	Contributes to unfavorable prognosis, promotes tumor progression	[141]
OC	Writer	METTL3	PTEN, PI3K, Akt, mTOR, miR-126-5p	Promotes the progression and tumorigenesis	[142]
		WTAP	MAPK, AKT	Contributes to worse survival outcome and promote tumor progression	[143]
	Eraser	FTO	cAMP signaling	Inhibits tumorigenesis and ovarian cancer stem cell self-renewal	[144]
		ALKBH5	NANOG	Promotes tumor progression	[145]
	Reader	YTHDF2	FBW7, BMF	Promotes tumor progression	[146]
		IGF2BP1	*SRF1, PDLIM7, FOXK1*	Promotes tumor progression and correlates with poor prognosis	[147]
		YTHDF1	*EIF3C*	Contributes to adverse prognosis, promotes tumorigenesis and metastasis	[148]
OS	Writer	WTAP	*HMBOX1*	Promotes osteosarcoma growth and metastasis	[149]
	Eraser	ALKBH5	YAP, pre-miR-181b-1	Suppresses cell growth, migration, invasion, and triggers cell apoptosis.	[150]
PAAD	Writer	METTL3	PHLPP2, Akt, miR-25-3p	Promotes the initiation and progression of cancer	[151]
		METTL14	*PERP*	Promotes cancer cell proliferation and migration	[152]
		WTAP	*WTAPP1*, Wnt signaling	Induces malignant phenotypes of cancer	[153]
	Eraser	FTO	*PJA2*	Suppresses the proliferation, invasion, and metastasis	[154]
		ALKBH5	PER1, YTHDF2	Inhibits cancer cell proliferation, migration, invasion, tumor growth	[155]
			*WIF-1*, Wnt pathway	Sensitizes to chemotherapy and inhibits cancer cell proliferation, migration and invasion	[156]
	Reader	YTHDC1	MiR-30d	Contributes to favorable prognosis, and represses pancreatic tumorigenesis	[157]
		IGF2BP2	LncRNA DANCR	Contributes to poor outcome and promotes cancer cell proliferation	[158]
PRAD	Writer	METTL3	GLI1	Promotes cell proliferation, survival, colony formation, and invasion	[159]
			MYC	Contributes to poor prognosis, promote development and progression of cancer	[160]
	Reader	YTHDF2	*LHPP, NKX3–1*	Contributes to poor prognosis and inhibit proliferation and migration of cancer	[38]
RB	Writer	METTL3	PI3K, AKT, mTOR, P70S6K, 4EBP1	Promotes tumor progression	[161]
RCC	Writer	METTL14	*BPTF*	METTL14 deficiency promoted RCC metastasis	[162]
	Eraser	FTO	SLC1A5	Contributes to the growth and survival of cancer cell	[163]
	Reader	IGF2BP3	*DMDRMR, CDK4*, *COL6A1, LAMA5, FN1*	Contributes to poor outcomes and promotes cell proliferation	[164]
TGCT	Writer	VIRMA	–	Contributes to tumor progression and cisplatin resistance	[165]

*EC* Endometrial cancer, *GSC* Glioblastoma stem cell, *HSCC* Hypopharyngeal squamous cell carcinoma, *LSCC* Laryngeal squamous cell carcinoma, *MEL* Melanoma, *NPC* Nasopharyngeal carcinoma, *NSCLC* Non-small-cell lung carcinoma, *OSCC* Oral squamous cell carcinoma, *TGCTs* Testicular germ cell tumors, *TNBC* Triple negative breast cancer

### m6A modification regulators function as tumor promoters

#### m6A modification regulators upregulate oncogenes

METTL3 promoted YAP translation by recruiting YTHDF1/3 and eIF3b, and increased YAP expression by the MALAT1/miR-1914-3p axis, leading to drug resistance and metastasis in non-small-cell lung carcinoma (NSCLC) [132]. METTL3 facilitated CRC progression by stabilizing *SOX2* [80], *HK2* and *GLUT1* [81] via an m6A-IGF2BP2/3 pathway, or by activating the GLUT1/mTORC1 axis in an m6A-dependent manner [79]. METTL14 enhanced BC proliferation and progression by increasing m6A modification and the expression of CXCR4 and CYP1B1 [166]. METTL14 blocked AML myeloid differentiation while promoting AML proliferation by upregulating MYB and MYC through m6A modification [56]. ALKHB5 upregulation by hypoxia decreased the m6A modification of *NANOG* mRNA and upregulated NANOG to induce the phenotype of BC stem cells [167]. ALKBH5 promoted the tumorigenicity and self-renewal of GBM stem-like cells by maintaining FOXM1 expression though demethylating FOXM1 nascent transcripts [98]. FTO-induced m6A demethylation decreased YTHDF2-mediated mRNA decay of programmed cell death protein 1 (PD-1), CXCR4, and SOX10, which enhanced melanoma tumorigenesis and anti-PD-1 resistance [137]. YTHDF3 enhanced the translation of m6A-marked ST6GALNAC5, GJA1 and EGFR, leading to brain metastasis of BC [70].

#### m6A modification regulators downregulate tumor suppressor genes

METTL3 inhibited the expression of SOCS2 via an m6A/YTHDF2 mechanism, which resulted in HCC tumorigenicity and metastasis [111]. METLL3 was downregulated in sorafenib-resistant HCC, and METTL3 inhibition conferred autophagy-related sorafenib resistance in HCC by decreasing the expression of FOXO3 in an m6A/YTHDF1 manner [168]. The METTL3/YTDHF2 axis has been found to induce β-catenin and PCNA upregulation by inhibiting the expression of YPEL5, which enhances tumorigenicity and metastasis in CRC [169]. METTL4 promoted the growth and metastasis of PAAD by decreasing the expression of PERP via m6A modification [152]. METTL14 inhibited skin tumorigenesis by promoting global genome repair through DDB2 m6A methylation and YTHDF1-mediated translation [170]. WTAP promoted the posttranscriptional suppression of Ets proto-oncogene 1 (Ets-1), contributing to HCC progression though the HuR/Ets-1/p21/p27 pathway [113]. ALKBH5 promoted the development and maintenance of AML and enhanced the self-renewal of leukemia stem/initiating cells by downregulating TACC3 [60]. YTHDF2 promoted the decay of UBXN1 mRNA via METTL3-mediated m6A modification, which induced the activation of the NF-κB pathway and promoted glioma progression [171].

### m6A modification regulators function as tumor suppressors

#### m6A modification regulators downregulate oncogenes

METTL14 inhibited CRC proliferation and metastasis by downregulating the oncogenic lncRNA XIST in an m6A-YTHDF2 manner [51]. The METTL14/YTHDF2 axis decreased *SOX4* mRNA expression and inhibited CRC epithelial to mesenchymal transition (EMT) and metastasis [84]. METLL14 was downregulated in RCC and led to the stability of bromodomain PHD finger transcription factor (BPTF) via m6A modification, which promoted metastasis and glycolytic reprogramming in RCC [162]. Downregulation of METTL3 and METTL14 increased the expression of TRIM7 via an m6A-YTHDF2 manner and thus promoted OS tumorigenesis and chemoresistance by the ubiquitination of breast cancer metastasis suppressor 1 (BRMS1) [172]. ALKBH5 impaired the stability of LYPD1 through an m6A-IGF2BP1 mechanism and inhibited the malignant behaviors of HCC [116]. FTO reduced the m6A modification and the stability of PDE1C and PDE4B that promoted cAMP hydrolysis, which inhibited cAMP signaling and suppressed the self-renewal of ovarian cancer stem cells [144]. YTHDF2 can inhibit HCC proliferation and growth by binding to m6A-modified EGFR mRNA and degrading EGFR in HCC cells [173]. YTDHC2 promoted the decay of SLC7A11 in an m6A-dependent manner and consequently suppressed the tumorigenesis of lung adenocarcinoma by targeting SLC7A11-mediated antioxidant function [174].

#### m6A modification regulators upregulate tumor suppressor genes

METTL14 inhibited CRC progression by regulating the processing of miR-375 that targeted YAP1 and SP1 [83]. METTL14 interacted with DGCR8 to suppress the metastasis of HCC by promoting the processing of miR-126 in an m6A-dependent manner [112]. ALKBH5 promoted the expression of PER1 in an m6A-YTHDF2 manner and inhibited PAAD progression by reactivating the ATM-CHK2-P53/CDC25C pathway [155]. ALKBH5 prevented the progression and increased the sensitivity of BLC to cisplatin via the m6A-casein kinase 2 (CK2) α-glycolysis pathway [175]. FTO decreased the m6A modification of praja ring finger ubiquitin ligase 2 (PJA2) and upregulated PJA2 to inhibit the Wnt pathway, thereby suppressing the progression of PAAD [154]. m6A modification was decreased in ocular melanoma due to METTL3 downregulation and ALKBH5 upregulation, which promoted YTHDF1-mediated translation of histidine triad nucleotide-binding protein 2 (HINT-2), a tumor suppressor of ocular melanoma [138].

## Drug discovery of m6A modulators

### Natural products from traditional medicines targeting m6A regulators

Natural products from traditional medicine could be used as a chemical library for m6A-targeting anticancer drug discovery. In this section, we focus on mechanistic insight into natural products derived from traditional medicines targeting m6A regulators and their current findings in cancer treatment.

#### Phenols

**Fig. 3:**
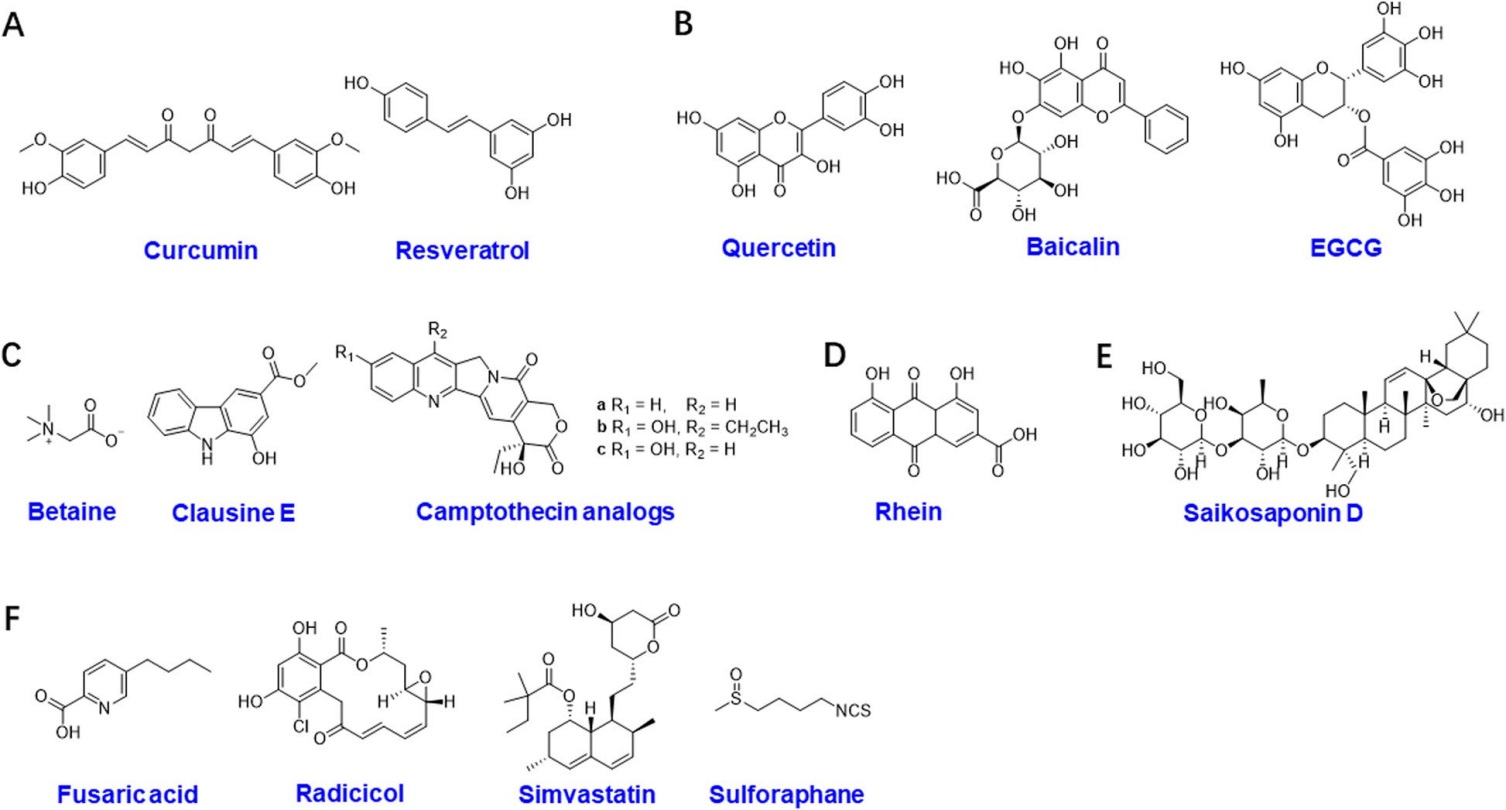
The chemical structures of natural products regulating m6A modification. **A** Phenols, **B** flavonoids, **C** alkaloids, **D** anthraquinone, **E** terpenoids, and **F** other natural products

Curcumin (Fig. 3A), a natural phenolic compound, reduced the expression of ALKHB5 and induced higher m6A-modified TNF receptor-associated factor 4 (TRAF4) mRNA that was bound by YTHDF1, leading to enhanced translation of TRAF4 [176]. Resveratrol (Fig. 3A) is a natural polyphenol with antioxidant, anti-inflammatory, heart-protective and anticancer properties [177]. Combining resveratrol with curcumin effectively improves growth performance and intestinal mucosal integrity by decreasing m6A as evidenced by enhanced YTHDF2 in the ileum [178].

#### Flavonoids

Quercetin (Fig. 3B) is commonly used as a dietary supplement and has many biological functions, including anticancer activities. Fluorescence quenching measurements indicated that among the 3 flavonoids (quercetin, apigenin, and naringenin), quercetin showed the strongest binding with FTO through hydrophobic interactions and hydrogen bonds [179]. In addition, quercetin has a synergistic effect with cisplatin on inhibiting the proliferation, migration and invasion of HeLa and SiHa cells by inhibiting the expression of METTL3 [180]. Baicalin (Fig. 3B) is widely found in the traditional Chinese medicine (TCM) Huang Qin, and it possesses significant antitumor effects in many cancers [181]. The baicalin hydrate inhibited tumor growth in NPC both in vivo and in vitro by influencing the genomic stability and affecting the splicing of Suv39H1 by upregulating m6A RNA methylation, as evidenced by increased *METTL3* and *METTL14* and decreased *FTO* and *ALKBH5* [182]. Epigallocatechin gallate (EGCG, Fig. 3B) is a tea flavonoid with powerful antioxidant, anti-inflammatory and anticancer effects, which may be associated with the regulation of cyclin A2 and CDK2 in an m6A-dependent manner mediated by inhibiting the expression of FTO and increasing expression of YTHDF2 [183].

#### Alkaloids

Betaine (Fig. 3C) is rich in the roots of *Beta Vulgaris* and acts as a methyl donor in the transformation of homocysteine to methionine [184]. As methionine is a substrate for SAM, an essential methyl group donor for mRNA m6A modification, betaine is likely to play an important role in m6A methylation. Zhang et al. found that betaine suppressed the expression of the m6A methylases METTL3 and METTL14 but facilitated the expression of the demethylases FTO and ALKBH5 in HepG2 cells [185]. In addition, clausine E (Fig. 3C) and camptothecin and their analogs (Fig. 3C) exhibited direct FTO-targeting bioactivity [186, 187]. Among them, clausine E dose-dependently inhibited the demethylation activity of FTO with an half maximal inhibitory concentration (IC_50_) value of 27.79 μM [186]. Meanwhile, clausine E inhibited FTO with a dissociation constant Kd value of 4.59 ± 1.51 μM, and the binding constant Ka (L mol^− 1^) between camptothecin and FTO was 3.74 × 10^− 4^ [186, 187].

#### Anthraquinone

Rhein (Fig. 3D), an anthraquinone rich in *Rheum rhabarbarum* [188], was identified as the first cell-active reversible and competitive inhibitor of FTO [189, 190]. Molecular modeling combined with biophysical techniques revealed that the inhibition of FTO by rhein occurred through directly binding to nucleic acids, competitively binding to the 2-oxoglutarate (2-OG) cofactor at the active site, or both [189].

#### Terpenoids

Saikosaponin is a classical triterpenoid that is extracted from *Radix Bupleuri* (Chinese name: Chaihu) and possesses anti-inflammatory and anticancer activities [191]. Saikosaponin D (Fig. 3E) inhibited FTO to rescue m6A hypomethylation in MYC and RARA. These actions in turn disrupted the stability of MTHFR and BCL2, thus sensitizing MV4–11- or Kas-1-resistant human myeloid mononuclear leukemia cells to tyrosine kinase inhibitors [192].

#### Other natural products targeting m6A regulators

Apart from the natural products mentioned above, other active natural products have been shown to possess biological activities against m6A and to exert anticancer activity. Fusaric acid (Fig. 3F) is a mycotoxin produced by *Fusarium* species [193]. It caused a decrease in p53 expression in HepG2 cells by downregulating m6A methylation of p53 mRNA, as indicated by the decreased expression of METTL3 and METTL14. In addition, the translation of p53 was simultaneously blocked by downregulating YTHDF1, YTHDC2, and YTHDF3 [194]. Radicicol (Fig. 3F) was isolated from the fungus *Monosporium bonorden* [195]. The crystal structure showed that the 4-Cl-1,3-diol group was an essential structure in radicicol responsible for binding to the FTO protein with an IC_50_ value of 16.04 μM [196]. Simvastatin (Fig. 3F) is a synthetic modification of a fermentation product derived from *Aspergillus terreus* [197], and it inhibited the migration and invasion of A549 cells by reducing m6A enrichment and its methyltransferase METTL3 in EZH2 mRNA, thus inhibiting the interaction between IGF2BP2 and EZH2 [198]. Sulforaphane (Fig. 3F) was identified as an epigenetic modulator by diminishing m6A methylation levels in BC cells to induce cell cycle arrest, autophagy and apoptosis [199].

### Lead compounds targeting m6A regulators from integrating AI technology and chemosynthesis

Modern approaches that integrate AI technology and chemosynthesis into the field of drug discovery have advantages such as speed, ease of use, and cost saving. Here, we summarize the anticancer m6A modulators that have been discovered with the help of modern technologies in recent years.

#### Targeting demethylases

**Fig. 4:**
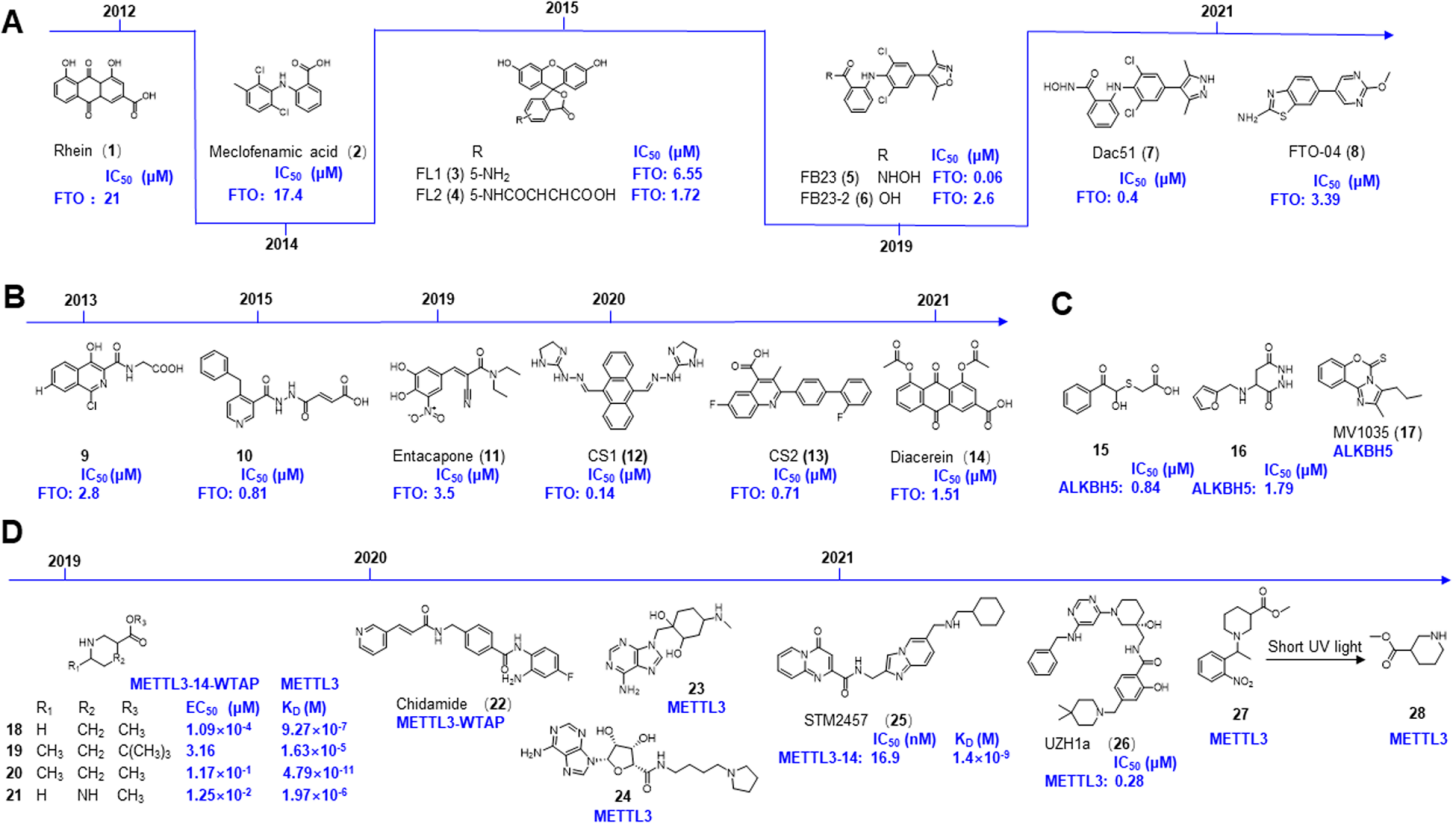
The chemical structures of lead compounds targeting m6A regulators from AI-based approaches. **A** Compounds targeting FTO derived from MA. **B** Compounds targeting FTO derived from the structure-based strategy. **C** Compounds targeting ALKBH5. **D** Compounds targeting METTL3

Since FTO was the first recognized m6A modification demethylase, targeting FTO is currently the most popular direction in research on m6A regulation. Cai-Guang Yang and coworkers developed a series of FTO inhibitors by applying AI-based approaches. The crystal structure of FTO was used in docking studies to screen the inhibitor of FTO from the drug-like SPECS database that contains 100,000 compounds. The natural product rhein (**1**; Fig. 4A) was identified as the first cell-based FTO inhibitor, which also inhibited ALKBH2 activity with a IC_50_ value on the same order of magnitude as FTO (IC_50_ = 21 μM) in 2012 [189]. To avoid competition with the AlkB subfamily, a high-throughput fluorescence polarization (FP) assay was applied to screen selective inhibitors of FTO from an older drug library containing 900 drugs. The anti-inflammatory drug meclofenamic acid (**2**; Fig. 4A) was identified as an inhibitor of FTO (IC_50_ = 17.4 μM) instead of ALKBH5 in 2014 [200]. Furthermore, 8 fluorescein molecules, which have structures similar to those of **2**, were designed and synthesized. The structure-activity relationships of these fluorescent FTO inhibitors are elucidated through the X-ray crystal structures of FTO/fluorescein complexes. Among these fluorescein derivatives, FL1 (**3**) and FL2 (**4**) (Fig. 4A) were selected as bifunctional molecules for selectively inhibiting and specifically photoaffinity labeling of FTO with IC_50_ values of 6.65 and 1.72 μM in HeLa cells, respectively [201]. In 2019, Yang et al. employed a structure-based rational design and achieved a promising FTO inhibitor FB23 (**5**) and it inhibited FTO-mediated demethylation with an IC_50_ value of 0.06 μM, which is 100-fold more active than that of **2**. Due to the poor permeability of 5 against AML cells, FB23–2 (**6**; Fig. 4A) with significantly improved antiproliferative activity and cellular efficacy was synthesized. Both **5** and **6** display high selectivity toward FTO but no effect on ALKBH5 demethylation in vitro. Mechanistically, 6 directly bound to and inhibited FTO, upregulating the expression of RARA and ASB2 and downregulating the expression of MYC and CEBPA to exert antileukemia therapeutic effects on a series of AML cell lines, patient-derived primary leukemia cells and patient-derived xenograft (PDX) mouse models [202]. In 2021, Yang et al. published a new finding that tumors exploit FTO-mediated regulation of glycolytic metabolism to evade immune surveillance. They developed a new potent FTO inhibitor Dac51 (**7**; Fig. 4A) based on 5/6. It exerted promising inhibitory activity on FTO and inhibits the glycolytic capacity of B16-OVA and LLC cell lines via the FTO-m6A-Jun/Cebpb signaling pathway [203]. Moreover, **7** may exert antitumor effects mediated by T cells to prevent tumors from recurring through the memory T cell response in patient-derived organoids and a mouse model of diverse cancer types. Impressively, a combination treatment of **7** and anti-programmed death-ligand 1 (PD-L1) blockade could enhance therapeutic outcomes [203]. Inspired by the binding sites of FTO in **2**, a combination of structure-based drug design and molecular docking with Schrödinger software was applied to screen FTO inhibitors, FTO-02 (IC_50_ = 2.2 μM) and FTO-04 (**8**; IC_50_ = 3.39 μM). However, only the anticancer ability of **8** was validated; it was found to inhibit neutrosphere formation in multiple GSC cell lines and significantly increased m6A levels [204]. This work represented an important step forward by combining structure-based drug design and a high-throughput in vitro inhibition assay system to identify a new chemical class of FTO inhibitors with tightly defined physicochemical properties.

FTO is a 2-OG-dependent N-methyl nucleic acid demethylase, and approximately 150 2-OG analogs are screened by differential scanning fluorometry- and liquid chromatography-based assays [205], among which **9** (Fig. 4B) have been used in clinical studies and have also shown inhibitory activity against FTO [205]. Furthermore, **10** (Fig. 4B) was generated with distinct selectivity for FTO (IC_50_ = 0.81 μM) against other AlkB subfamilies and 2-OG oxygenases [206]. A prototype example of AI-based approaches was applied in the discovery of the FTO inhibitor entacapone (**11**; Fig. 4B) from a library of FDA-approved drugs. Huang et al. combined multiple methodologies, including structure-based hierarchical virtual screening strategies, biochemical experiments, in vivo experiments, and transcriptome sequencing analyses, to identify entacapone as an FTO inhibitor with an IC_50_ value of 3.5 μM [207]. In 2020, through structure-based virtual screening, Chen et al. found two potent FTO inhibitors, CS1 (**12**) and CS2 (**13**) (Fig. 4B). They shared similar key biological pathways with 6, which directly bound to FTO and efficiently suppressed its m6A demethylase activity, with IC_50_ values of 142.6 nM and 712.8 nM, respectively. Nonetheless, 12 and 13 targeting FTO might exert antileukemic activity by suppressing AML stem cell maintenance, sensitizing cancer cells to T cell cytotoxicity, and overcoming immune evasion [59]. Diacerein (**14**; Fig. 4B) was another potent FTO inhibitor identified by a single quantum dot-based fluorescence resonance energy transfer (FRET) sensor. Rather than being a chelator of metal ions or a structural mimic of 2-oxyglutarate, diacerein directly bound to FTO (IC_50_ = 1.51 μM) to inhibit the demethylation activity of FTO in HeLa cells [208].

The FTO inhibitors mentioned above provide small molecular tools for the study of m6A modification and the biological function of FTO. In addition, other potent m6A demethylase ALKBH5 inhibitors, such as **15** and **16**, were identified from a library of 144 000 compounds, and most of them showed strong anticancer properties [209–211]. Among them, MV1035 (**17**; Fig. 4C), selected by 3D proteome-wide scale screening, was recently recognized as a ALKBH5 inhibitor that possessed potent anticancer activity against U87 glioblastoma cells [211]. Miao et al. found that the HSP90 inhibitor ganetespilb facilitates the translation of DNAJB4 by m6A modification at A114 site by increasing the expression of YTHDF3 in M14 cells [212].

#### Targeting methylases

As epitranscriptomic writers for m6A methylation, the m6A methylases METTL3 is involved in various stages of multiple hematoma and solid malignancies, including tumor stemness, immune microenvironment, drug resistance, metastasis and recurrence [85, 96, 132]. Therefore, METTL3 has been recognized as one of the most promising therapeutic targets for anticancer drug discovery. In an effort to explore a specific inhibitor of METTL3, a virtual screening of ZINC and DrugBank 4.0 databases identified 4 compounds (**18**–**21**; Fig. 4D) with piperidine or piperazine rings, and they functioned as METTL3/14-WTAP activators to facilitate RNA methylation, which increased the mRNA m6A levels to shift the cell cycle to G_0_ and G_1_ phase without cytotoxic effects at 100 μM [213]. Another METTL3 and WTAP inhibitor was chidamide (**22**; Fig. 4), which downregulates c-MET expression by suppressing m6A methylation, as evidenced by the downregulation of METTL3 and WTAP in NSCLC to increase the therapeutic efficacy of crizotinib [214]. Meanwhile, by screening a library of 4000 analogs and derivatives of the adenosine moiety of SAM accompanied by the high-throughput docking assay, two adenine derivatives, **23** and **24** (Fig. 4) were selected as METTL3 inhibitors. They have been validated to bind to METTL3 by X-ray crystallography [215]. In addition, a high-throughput screen of 250,000 diverse drug-like compounds was performed. Among these candidates, STM2457 (**25**; Fig. 4) was validated to bind to the METTL3-METTL14 heterodimer specifically and directly in the SAM site by X-ray crystallography. As the first METTL3 inhibitor, **25** has been validated to increase cell differentiation and apoptosis by reducing m6A enrichment in METTL3-dependent core leukemogenic m6A substrates, such as *HOXA10* and *MYC*. As a result, 25 exerts a potent therapeutic effect on multiple AML mouse models by affecting the AML stem cell or leukemia propagating compartment [9]. The effects of STM2457 in other tumor types are now being investigated by STORM, which is aiming to put STM2457 in phase trials in 2022 [8]. By screening an adenine-based library with a homogenous time-resolved fluorescence (HTRF) enzyme inhibition assay, a potent and selective METTL3 inhibitor, UZH1a (**26**; Fig. 4) was identified. It was further validated to selectively bind to METTL3 by X-ray crystallography, and it slightly suppresses the expression of METTL3 but significantly reduces m6A levels in the mRNA fraction in the leukemia cell line MOLM-13 and human osteosarcoma U2OS cells [216].

In contrast to conventional inhibitors, photoactivated compounds have been creatively constructed. With the assistance of computational docking, Lan et al. identified a caged molecule activator of METTL3/14, photocaging substituent-linked MPCH (**27** and **28**) (Fig. 4). It activates METTL3/14 and results in considerable m6A hypermethylation after short UV light exposure in different cells. Owing to the rapid uncaging of MPCH by light radiation, the side effects are minimal and can be controlled. As the release of medicine could be swiftly initiated by short light irradiation, it might be suitable for utilization in living systems instead of depending on the addition or deletion of endogenous enzymes [217]. The above small molecules targeting METTL3 showed potent therapeutic effects in tumor treatment, indicating that METTL3 could be the most promising target. However, since METTL3 is widely involved in the expression of various genes, the in vivo side effects of METTL3-targeted agents should be strictly tested. In addition to the inhibitors of METTL3 mentioned above, some other METTL3 inhibitors are now being investigated by Accent Therapeutics and Gotham Therapeutics. These companies are aiming to put their own METTL3 inhibitors into phase I trials in 2022 [8].

## Conclusions and perspectives

Epigenetic regulation has become a hot topic in recent decades and RNA m6A modification in cancer research has been developed into one of the most popular fields in recent years. Epigenetic regulation inhibitors, such as azacytidine and decitabine, which are two inhibitors of DNA methylation, have shown great anticancer effects in clinical use. The dysregulation of m6A modification frequently occurs in many types of cancers and m6A modification regulates the malignant phenotypes and behaviors primarily by controlling the expression of oncogenes and tumor suppressor genes. Notably, aberrant m6A modification is critically associated with tumor progression and cancer patient prognosis. Therefore, targeting m6A modification regulators might also be a potential and promising therapeutic strategy for cancer treatment.

There are many advantages in using traditional medicines or natural products to screen the inhibitors and activators of m6A medication regulators. First, the efficacy and safety of traditional medicines and natural products have been validated by generations through the repeated experiences of countless rounds of trial and error over thousands of years. Moreover, many bioactive small molecules derived from traditional medicines and natural products have novel chemical structures and multiple biological activities, and more than 60% of anticancer drugs are natural origins or contain the pharmacophores of natural products [218]. These powerful advantages make traditional medicines and natural products reliable sources for the discovery of new therapeutic agents targeting m6A modification.

**Fig. 5:**
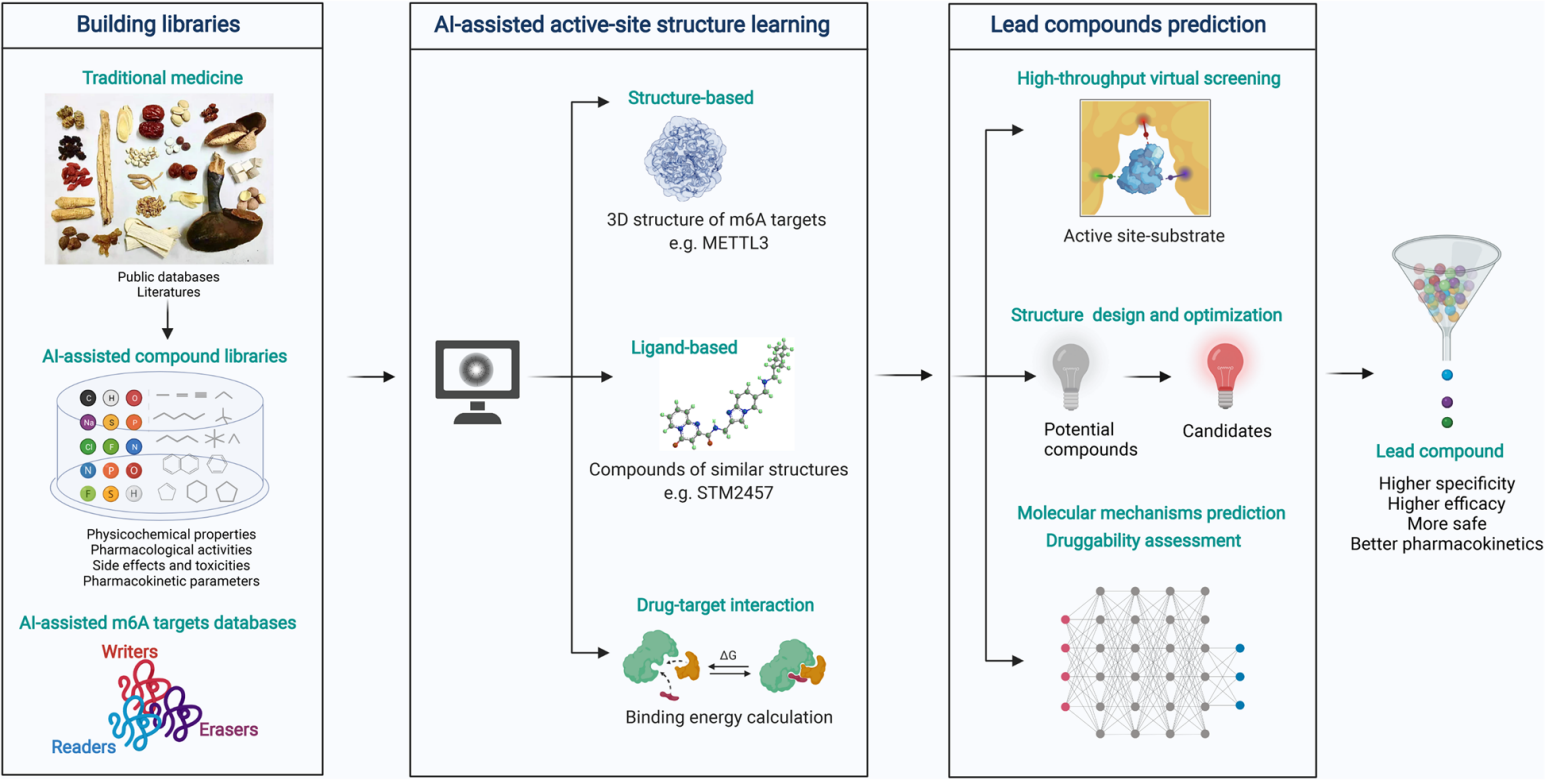
A framework for the m6A-targeting drug discovery through integration of AI and traditional medicines and natural products. The data collected and organized from protein databases and compound libraries were performed with high-throughput virtual screening, followed by screening and predication, modification and optimization of chemical structures, and assessment of druggability of lead compounds that target m6A modification

AI-assisted techniques have been widely used for the discovery and development of drug candidates [219, 220] and several online databases related to traditional medicines or natural products have been developed. The TCM Systems Pharmacology Database and Analysis Platform (TCMSP), is a comprehensive phytochemical database for drug discovery from herbal medicines, and it includes 29,384 ingredients of approximately 500 Chinese herbal medicines, more than 3, 000 targets, and 837 related diseases [221]. Indian Medicinal Plants, Phytochemistry, And Therapeutics (IMPPAT) is a database containing 9596 phytochemicals, 1742 Indian medicinal plants, and 1124 therapeutic uses spanning 27,074 plant-phytochemical associations and 11,514 plant-therapeutic associations [222]. Therefore, it would be more efficient to develop novel and effective therapeutic agents that inhibit m6A modification-mediated tumor progression by combining traditional medicines and natural product databases with AI-based drug discovery approaches. Additionally, we present a framework for m6A-targeting drug discovery through integration of AI and traditional medicines and natural products (Fig. 5). Specifically, we could collect and organize data from protein databases related to m6A modification regulators, and compound libraries (e.g., TCMSP, IMPPAT) that contain the natural origins, chemical structures, physicochemical properties, pharmacological activities, side effects and toxicities, and pharmacokinetic parameters of natural products and active small molecules derived from traditional medicines. Then, AI-based methods were used for high-throughput virtual screening of lead compounds through target protein structure-based approaches (e.g., molecular docking simulation), ligand-based approaches (e.g., quantitative structure-activity relationship (QSAR) models) and drug-target interaction data. With a range of AI techniques, we can better screen and predict potential compounds, develop the modification and optimization of chemical structures, and assess the druggability of lead compounds targeting m6A modification.

Although more than 20 m6A modification regulators have been identified, only a few of them have been confirmed to be druggable and could serve as therapeutic targets for cancer treatment. Meanwhile, many inhibitors and activators of m6A modification have been reported, but none of them have been approved for cancer treatment in the clinic. The currently developed m6A modification inhibitors and activators might have poor target specificity, therapeutic efficacy, safety, and pharmacokinetics [202, 204]. It takes several years, or even decades, to develop an anticancer drug from the laboratory to the clinic and incurs high costs. The current m6A modification inhibitors and activators must be investigated thoroughly in a series of preclinical and clinical trials before approval for clinical use. These obstacles critically hinder the development of current m6A modification inhibitors and activators into drugs for clinic use. Currently, revolutionary AI-assisted approaches to drug discovery, design, and development have been developed, and we could take full advantages of AI to develop m6A inhibitors and activators with better specificity, efficacy, safety, and pharmacokinetics, which will reduce the cost and shorten the time of drug development related to m6A modification by AI. We therefore believe that an increasing number of novel, specific, and effective m6A modification inhibitors and activators will be developed and approved for clinical use in the near future.

## Data Availability

Not applicable.

## Abbreviations

AI: Artificial intelligence
AK4: Adenylate kinase 4
ALKBH5: AlkB homologue 5
AML: Acute myeloid leukemia
BC: Breast cancer
BLC: Bladder cancer
BPTF: Bromodomain PHD finger transcription factor
BRMS1: Breast cancer metastasis suppressor 1
CK2: Casein kinase 2
CRC: Colorectal cancer
EC: Endometrial cancer
EGCG: Epigallocatechin gallate
eIF3: Eukaryotic initiation factor 3
EMT: Epithelial to mesenchymal transition
Ets-1: Ets proto-oncogene 1
FMR1: Fragile X mental retardation 1 gene
FMRP: Fragile X mental retardation protein
FP: Fluorescence polarization
FRET: Fluorescence resonance energy transfer
FTO: Fat mass and obesity-related protein
GBM: Glioblastoma
GC: Gastric cancer
GSC: Glioblastoma stem cell
HCC: Hepatocellular carcinoma
HINT-2: Histidine triad nucleotide-binding protein 2
HNRNP: Heterogeneous nuclear ribonucleoprotein
HNSCC: Head and neck squamous cell carcinoma
HTRF: Homogenous time-resolved fluorescence
IC_50_: Half maximal inhibitory concentration
IGF2BPs: Insulin-like growth factor 2 mRNA-binding proteins
IMPPAT: Indian Medicinal Plants, Phytochemistry, And Therapeutics
LC: Lung carcinoma
LSCC: Laryngeal squamous cell carcinoma
m6A: N6-methyladenosine
MEL: Melanoma
METTL3: Methyltransferase-like 3
mRNAs: Messenger RNAs
MM: Multiple myeloma
NPC: Nasopharyngeal carcinoma
NSCLC: Non-small-cell lung carcinoma
OC: Ovarian cancer
2-OG: 2-oxoglutarate
OS: Osteosarcoma
OSCC: Oral squamous cell carcinoma
PAAD: Pancreatic adenocarcinoma
PD-1: programmed cell death protein 1
PD-L1: Programmed death-ligand 1
PDX: Patient-derived xenograft
PJA2: Praja ring finger ubiquitin ligase 2
PRAD: Prostate adenocarcinoma
pre-mRNAs: Precursor mRNAs
pri-miRNAs: Primary miRNAs
PRRC2A: Proline rich coiled-coil 2A
QSAR: Quantitative structure-activity relationship
RB: Retinoblastoma
RBM15: RNA binding motif protein 15
RCC: Renal cell carcinoma
SAM: S-adenosylmethionine
TCM: Traditional Chinese medicine
TCMSP: TCM Systems Pharmacology Database and Analysis Platfor
TGCTs: Testicular germ cell tumors
TNBC: Triple negative breast cancer
TRAF4: TNF receptor-associated factor 4
VIRMA: Vir-like m6A methyltransferase-associated
WTAP: Wilms tumor 1 associated protein
ZCCHC4: Zinc finger CCHC-type containing 4
ZC3H13: Zinc finger CCCH-type containing 13
